# Everybody's Page

**Published:** 1889-04-20

**Authors:** 


### Everybody's Page.

Some time ago the proprietor of a village hotel advertised for summer boarders. He was much surprised to receive a letter from a City gentleman asking him to state his terms, and send a sample of the drinking water of the place for analysis. Evidently the intending lodger had found out, probably by sad experience, that the purest of country air will not make up for impure water.

M. Dubois, a Nantes doctor, says that the pain of burns may be relieved by allowing the contents of a syphon of seltzer water to flow over the affected parts. He believes that this treatment not only gives immediate relief, but hastens the final cure, and ascribes the good effects to the carbonic acid gas, which "aerates" the seltzer, and to the lowering of the temperature of the burnt parts.

The Japanese have a species of sacred nut which they burn in joss-houses, and which has an odour that is supposed to be especially pleasing to the gods. The plant which bears it grows under water, and has a leaf and flower not unlike a pond lily. The nuts themselves are shaped somewhat like an ox's head, horns and all; indeed, at first sight, one would think they were carved. They are hard and tasteless when raw, but when cooked they resemble boiled chestnuts in flavour.

Professor Bergmann some time ago performed a rather uncommon surgical feat. Two cases came to him simultaneously for operation; one required amputation of the hip joint, the other excision of diseased bone-tissue from the humerus. The Professor did the amputation first, and used a part of the femur he had cut off to fill up the space left by the removal of the dead bone in the other patient's arm. Perfect union took place, but one cannot help asking, to whom does that arm rightly belong?

Automatic machines have been introduced into Brooklyn which are a great improvement on our London ones. Each box contains a supply of stamps, post-cards, pencils, and envelopes. It is divided into compartments for each of these articles, with a slot over the respective drawers in which to drop the coins; and as they are shaped like writing-desks, of convenient height, and are affixed to lampposts, a forgetful or unmethodical man may conduct all his correspondence in the street at any hour of the day or night.

Kola, in spite of its undoubtedly valuable properties, does not seem to be in great demand in Europe. It is not pleasanter to the taste than tea, coffee, or cocoa, and could hope to rival them only if it were cheaper; and it is not likely ever to become so, for in Africa the natives esteem it so highly that they are willing to give large prices for it. Adolf Krause, the African traveller, says that even in Africa Europeans eat kola only when they can get nothing else, and the attempt to introduce kola chocolate into this country has not been very successful.

In a book entitled "Practical Education," Mr. Charles G. Leland gives advice that will seem strange to many pedants and dogmatists. Put briefly, his idea of education is the extension and development of Froebel's system, now confined to the kindergarten, to the whole of school life—the training of the eye and hand by practical dealing with form and colour. The experiment has been tried, and with successful results, in a large school in Philadelphia. The devotees of "book-learning" will probably despise it, but this training of the faculties of observation, and development of manual skill, should certainly be of practical use in after life.

The Italians speak of a man who is so eccentric as to touch the verge of insanity without quite crossing the border, as "one who has a longer beard than Moses." Why the great Hebrew leader, one of the shrewdest and sanest of men, should be held akin to lunatics it is difficult to guess.

The Duallas—the strong, clever, and cheerful inhabitants of the Cameroons—carry out the principle of the "survival of the fittest" with Spartan rigour. Every child born among them is, at the age of five days, immersed in the river, and this treatment is continued daily till it is old enough to bathe itself or has succumbed to the process.

What is the origin of the phrase "to be led by the nose"? This: In India, where goods are carried on the backs of elephants, a carrier, in order to save the expense of employing several drivers, and to keep his train of animals in order, fastens the trunk of each elephant to the train of the animal in front of him. When the start is made the driver has only to look after the leader. When he moves the others are bound to follow—"led by the nose."

Even pure water, if too constantly used, will act as an irritant to the skin. One form of water cure, once popular, was to envelop the patient in a stratum of wet clothes, evaporation being prevented by an outer wrap of waterproof. This resulted in the production of an ugly eruption, which was supposed to be a diseased "humour of the blood" finding its way to the surface. As a matter of fact, the eruption only showed that the skin had been irritated by a mechanical agent, and a result quite as beneficial to health would have been obtained if the patient or his attendant had scratched the skin all over.

Salt is among the oldest of our manufactures. The brine-springs in Cheshire have been used for industrial purposes since the days of the Saxon kings. Droitwich, in Worcestershire, also is an ancient seat of the salt industry. "Wich" is the old name of all places where salt was manufactured, and still survives in the names "Northwich," "Nantwich,' and the like. In 816 Kenulph, king of the Mercians, gave "Hamilton, and ten houses in Wich," to the church at Worcester, and in 906 King Edwy bestowed on the same establishment "Jepstone, and five salt furnaces." A full account of the Cheshire "Wiches" is given in the Domesday Book, and we learn there the duties levied on the export of salt and the fines exacted for smuggling of any kind.

Those who have given close study to the requirements of the human system in the matter of food say that a healthy man requires 300 grains of nitrogen, and 4,600 grains of carbon daily to repair the waste that goes on during the twenty-four hours. As meat contains 100 grains of carbon and 300 of nitrogen in every 1,000 grains, it would be necessary to eat 6½lb. of meat to supply the amount of carbon the system requires, which would entail the consumption of a great deal more nitrogen than the system needs. On the other hand, bread contains 300 grains of carbon and 10 of nitrogen in every 1,000 grains, so that to obtain the amount of nitrogen required it is necessary to consume twice as much bread as will supply the requisite amount of carbon. These calculations indicate the value of a mixed diet. By consuming both animal and vegetable food a balance is struck between the superabundance of any substance contained in either; and it has been calculated that ¾lb. of meat and 2lb. of bread are sufficient to compensate for the daily waste in the system of a healthy man.

